# Supplementary material for: An imaging flow cytometry-based methodology for the analysis of single extracellular vesicles in unprocessed human plasma
Source: Commun Biol. 2022 Jun 29;5:633. doi: 10.1038/s42003-022-03569-5 (PMC9243126; doi:10.1038/s42003-022-03569-5)
Supplement: Supplementary file 2 — Supplemental Information [file 42003_2022_3569_MOESM2_ESM.pdf]

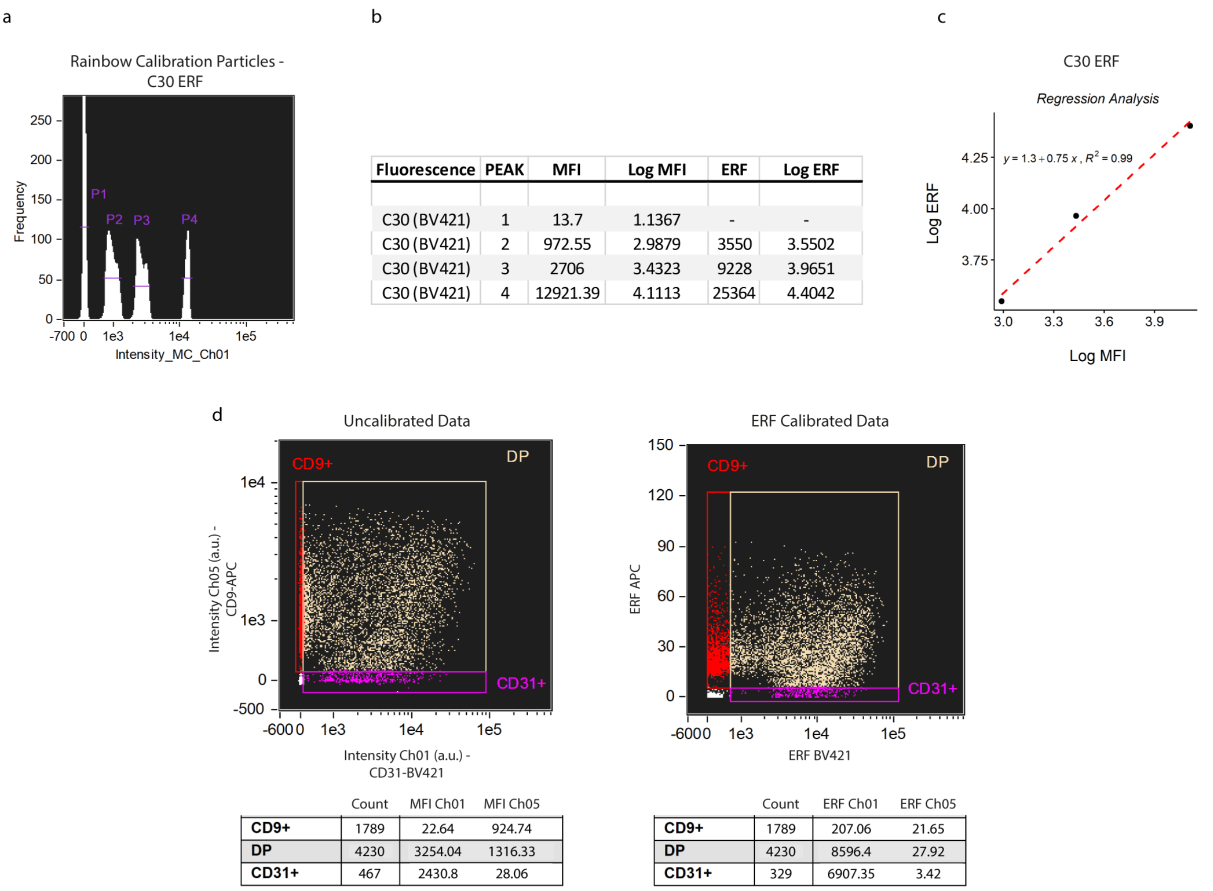

Fluorescence	PEAK	MFI	Log MFI	ERF	Log ERF
C30 (BV421)	1	13.7	1.1367	-	-
C30 (BV421)	2	972.55	2.9879	3550	3.5502
C30 (BV421)	3	2706	3.4323	9228	3.9651
C30 (BV421)	4	12921.39	4.1113	25364	4.4042

	Count	MFI Ch01	MFI Ch05
**CD9+**	1789	22.64	924.74
**DP**	4230	3254.04	1316.33
**CD31+**	467	2430.8	28.06

	Count	ERF Ch01	ERF Ch05
**CD9+**	1789	207.06	21.65
**DP**	4230	8596.4	27.92
**CD31+**	329	6907.35	3.42

Supplementary Figure 1 – Fluorescent calibration of BV421 **a**) The median fluorescent intensities (MFI) of each fluorescent peak of BV421 ERF calibration beads was measured with the same instrument/acquisition settings applied as used for EV acquisition. **b**) Calculation of the log of the MFI and ERF values (provided by the bead manufacturer). **c**) For both detection channels, the log of the MFI was plotted on the x-axis, and the log of the ERF values on the y-axis. A linear regression analysis was performed, respectively. **d**) Representative example of uncalibrated data (left) and corresponding ERF calibrated data (right).

Framework Criteria	What to report	Please complete each criterion
1.1 Preanalytical variables conforming to MISEV guidelines.	Preanalytical variables relating to EV sample including source, collection, isolation, storage, and any others relevant and available in the performed study.	From each of the 5 (human) healthy individuals , 12 mL of blood was collected (one drawing) into two BD Vacutainer® K3-EDTA-coated collection tubes (BD Biosciences, San Jose, USA). Whole blood was centrifuged (Heraeus Multifuge 1S) at 1910 x g for 10 minutes at room temperature. The plasma layer was then collected - leaving ~1 mm of plasma above the buffy coat - and centrifuged (Heraeus Fresco) at 16,000 x g for 10 minutes at room temperature. The resulting platelet-poor plasma (PPP) was divided into 700-μL aliquots in cryovials containing 28 μL of a 25x concentrated protease inhibitor cocktail solution (4% v/v) (cOmplete Protease inhibitor cocktail tablets, Roche, Mannheim, Germany) according to the manufacturers' instructions and stored at -80 °C.<br>All the procedures and animal housing conditions were carried out in strict accordance with current EU legislation on animal experimentation and were approved by the Institutional Committee for Animal Research (DEC protocol EMC No. AVD101002016635). Six weeks male C57BL/6J (JAX,GSP) mice (Jackson Labs, Bar Harbor, ME) were housed in Erasmus MC animal facility and housed in groups of 2-3/cage. They were maintained on a 12:12 h light-dark cycle and allowed ad libitum access to water and standard rodent food. The mice were anesthetized and blood (approximately 0.8 mL) was collected via the left ventricle using a 23-25 gauge needle. To ensure euthanasia of the animal post-procedure, mice were killed by cervical dislocation.
1.2 Experimental design according to MIFlowCyt guidelines.	EV-FC manuscripts should provide a brief description of the experimental aim, keywords, and variables for the performed FC	**1.1 Aim:** To develop an assay for the direct measurement of Extracellular Vesicles (EV) in unprocessed (human) plasma samples.<br>**1.2 Keywords:** Unprocessed Human Plasma; Extracellular Vesicles; Imaging Flow Cytometry; Quantify; Phenotype; Diagnostic Platform.<br>**1.3 Experiment variables:** Platelet-poor plasma (PPP) samples from 5 healthy individuals

	experiment(s) using MIFlowCyt checklist criteria: 1.1, 1.2, and 1.3, respectively. Template found at www.evflowcytometry.org.	and/or six week old male C57BL/6J (JAX,GSP) mice (Jackson Labs, Bar Harbor, ME) were stained with CFDA-SE, anti-tetraspanin antibodies (CD9, CD63, CD81) and CD31, and measured with Imaging Flow Cytometry (IFCM).

2.1 Sample staining details	State any steps relating to the staining of samples. Along with the method used for staining, provide relevant reagent descriptions as listed in MIFlowCyt guidelines (Section 2.4 Fluorescence Reagent(s) Descriptions).	**mAbs used:** The monoclonal antibodies (mAbs) used to stain human PPP were anti-CD9–APC, clone HI9a (6 µg/mL, BioLegend, San Diego, USA); anti-CD63–APC, clone H5C6 (200 µg/mL, BioLegend); and anti-CD81–APC, clone 5A6 (200 µg/mL, BioLegend. Human and mouse PPP were both stained with anti-human CD31–BV421, clone WM-59 (50 µg/mL, BioLegend) and anti-mouse CD31-APC, clone 390 (200 µg/mL, BioLegend). Isotype controls used were IgG1,k-BV421, clone MOPC-21 (100 µg/mL, BioLegend); IgG1,k-APC, clone MOPC-21 (200 µg/mL, BioLegend); and IgG2a,k-APC, clone RTK2758 (200 µg/mL, BioLegend).<br>**mAb preperation:** All mAbs were centrifuged for 10 minutes at 16,000 x g to reduce the number of (potential) mAb clumps. A volume of the top layer of each centrifuged mAb solution was carefully harvested (according to the dilutions needed, described below) and diluted in 0.22 µm-filtered PBS (fPBS) before being added to the samples.<br>**mAb pre-dilutions** All tetraspanin mAbs were diluted 30-fold in fPBS before staining (Final concentrations: CD9: 0.2 µg/mL, CD63: 6.6 µg/mL, CD81: 6.6 µg/mL); CD31-BV421 (anti-human) and CD31-APC (anti-mouse) were diluted 1000-fold (Final concentration: 50 ng/mL) and 62.5-fold (Final concentration: 3.2 µg/mL), respectively. The anti-tetraspanin antibody mixture was made by combining anti-CD9/anti-CD63/anti-CD81 in the same stock solution.<br>**CFDA-SE Stock solution preperation:** A carboxyfluorescein diacetate succinimidyl ester (CFDA-SE) stock solution was made with the Vybrant™ CFDA-SE Cell Tracer Kit from Invitrogen immediately prior to use according to the manufacturer's instructions: CFDA-SE powder was spun down using a table-top centrifuge, and 18 µL of dimethylsulfoxide (DMSO) was added. The mixture was thoroughly resuspended and incubated at room temperature for 10 – 15 minutes in the dark. The dissolved CFDA-SE was added to a total volume of 1.782 mL of fPBS to create a 50 µM CFDA-SE stock solution. Similar to the protocol used to prepare mAbs, this stock solution was centrifuged for 10 minutes at 16,000 x g to reduce potential CFDA-SE clumps; the top layer was carefully harvested before being added to the samples.<br>**Sample staining:** 30 uL of sample was added to a pre-defined volume of fPBS (dependant on

		the volume of mAb staining - total volume after mAb addition was set at 130 µL): 12.5 uL of the stock solutions containing mAbs labelled with –APC and 5 µL of the stock solutions containing mAbs labeled with –BV421 were added, resulting in the following concentrations used per test: anti-CD9 – 2.5 ng, anti-CD63 – 83 ng, anti-CD81 – 83 ng, anti-CD31 (anti-human) – 1 ng, anti-CD31 (anti-mouse) – 40 ng per test. Equivalent amounts of isotype control was used for each antibody.<br>Samples were then incubated overnight at 4 °C to ensure optimal saturation of the available EV epitopes; this incubation time was determined by adding the anti-tetraspanin antibody mix to fPBS and PPP samples and performing acquisition at set intervals (1/3/6 hours and O/N).<br>CFDA-SE labeling was performed on the day of data acquisition by adding 100 µL of the 50 µM CFDA-SE stock solution to the samples, followed by 30 minutes of incubation at room temperature in the dark. Control samples not stained with CFDA-SE were incubated with 100 µL fPBS instead. All samples were brought to a total volume of 380 µL using fPBS before IFCM measurements.

2.2 Sample washing details	State any steps relating to the washing of samples.	No sample washing was performed; background fluorescence induced by our protocol is described in detail in this work.
2.3 Sample dilution details	All methods and steps relating to sample dilution.	For sample staining (descibed above), 30 uL of sample was incubated O/N in a total volume of 130 uL, resuling in 4.25-fold sample dilution. This volume was topped-up with 250 uL fPBS to a total volume of 380 uL, resulting in a total dilution of ~12.6-fold. For serial dilution experiments, samples were diluted four times (4-fold each step) by mixing 100 uL of sample with 300 uL of fPBS.
3.1 Buffer alone controls.	State whether a buffer-only control was analyzed at the same settings and during the same experiment as the samples of interest. If utilized it is recommended that all samples be recorded for a consistent set period of time e.g. 5 minutes, rather than stopping analysis at a set recorded event count e.g. 100,000 events. This allows comparisons of total particle counts between controls and samples.	Buffer-only control of 0.22 µm-filtered PBS (fPBS) was recorded during the same experiment at the same imaging flow cytometer with acquisition settings similar to all other samples, including laser power and flow rate. All samples were recorded for 3 minutes to allow comparisons of total particle counts between controls and samples. In gerneral, <10 fluorescent events were acquired within this time period for each of the established gating regions.

3.2 Buffer with reagent controls.	State whether a buffer with reagent control was analyzed at the same settings, same concentrations, and during the same experiment as the samples of interest. If used state what the results were.	Buffer with reagent controls (single-stained with 12.5 µL anti-CD9 – 2.5 ng/test, 12.5 µL anti-CD63 – 83 ng/test, 12.5 µL anti-CD81 – 83 ng/test, 5 µL anti-CD31 (anti-human) – 1 ng/test, 5 µL anti-CD31 (anti-mouse) – 40 ng/ test, 100 µL of the 50 µM CFDA-SE stock solution) were recorded during the same experiment at the same imaging flow cytometer with acquisition settings similar to all other samples, including laser power and flow rate. All samples were recorded for 3 minutes to allow comparisons of total particle counts between controls and samples. In general, after 3 minutes, 600-700 fluorescent events (-APC) were recorded in buffer-control with anti-tetraspanin cocktail, <10 events in buffer-control with anti-CD9, ~100-200 events in buffer-control with anti-CD63, ~400-500 events in buffer-control with anti-CD81, ~<50 events in buffer-control with anti-mouse anti-CD31, ~200 events in buffer-control with anti-human anti-CD31 (-BV421), and <10 events in buffer-controls with CFDA-SE (CFSE)
3.3 Unstained controls.	State whether unstained control samples were analyzed at the same settings and during the same experiment as stained samples. If used, state what the results were, preferably in standard units.	Unstained control samples were measured at the same dilution as matched stained and isotype control samples, and were recorded during the same experiment at the same imaging flow cytometer with acquisition settings similar to all other samples, including laser power and flow rate. No substantial changes in fluorescence signal were observed between unstained and matched isotype controls.

3.4 Isotype controls.	The use of isotype controls is applicable to immunofluorescence labelling only. State whether isotype controls were analyzed at the same settings and during the same experiment as stained samples. If utilized, state which antibody they are matched to, the concentration used, and what the results were (Section 4.2, 4.3, 4.4). Due to conjugation differences between manufacturers if should be stated if the isotype controls are from the same manufacturer as the matched antibodies.	Isotype controls samples were measured at the same dilution and at the same concentration as matched stained controls and were recorded during the same experiment at the same imaging flow cytometer with acquisition settings similar to all other samples, including laser power and flow rate. No substantial changes in fluorescence signal were observed between unstained and matched isotype controls.<br><br>**Isotype - mAb matching:**<br>IgG1,k-BV421, clone MOPC-21 (100 µg/mL, BioLegend) matched with anti-human CD31–BV421, clone WM-59 (50 µg/mL, BioLegend);<br>IgG1,k-APC, clone MOPC-21 (200 µg/mL, BioLegend) matched with anti-CD9–APC, clone HI9a (6 µg/mL, BioLegend, San Diego, USA); anti-CD63–APC, clone H5C6 (200 µg/mL, BioLegend); and anti-CD81–APC, clone 5A6 (200 µg/mL, BioLegend)<br>IgG2a,k-APC, clone RTK2758 (200 µg/mL, BioLegend) matched with anti-mouse CD31-APC, clone 390 (200 µg/mL, BioLegend)<br>No isotype control for CFDA-SE was used.<br><br>All isotype controls are from the same manufacturer as the matched antibodies.

3.5 Single-stained controls.	State whether single-stained controls were included. If used state whether the single-stained controls were recorded using the same settings, dilutions, and during the same experiment as stained samples and state what the results were, preferably in standard units (Section 4.2, 4.3, 4.4).	Single-stained control samples were included for every mAb used in this work, and were measured at the same dilution and at the same concentration as matched stained controls and were recorded during the same experiment at the same imaging flow cytometer with acquisition settings similar to all other samples, including laser power and flow rate. Single-stained controls aided in the establishment of the compensation matrix (to eliminate spectral overlap between detection channels).The following results were obtained for a representative single-stained PPP sample:**anti-CD9, anti-CD63, anti-CD81 (mix)** Counts: 7666, Median Fluorescent Intenstiy: 906, Equivalent number of Reference Fluorophores: 52**CFSE** Counts: 3234, Median Fluorescent Intenstiy: 816, Equivalent number of Reference Fluorophores: 134**anti-human anti-CD31**Counts: 3341 Median Fluorescent Intenstiy: 4101, Equivalent number of Reference Fluorophores: 12701
3.6 Procedural controls.	State whether procedural controls were included. If used, state the procedure and if the procedural controls were acquired at the same settings and during the same experiment as stained samples.	No procedural controls were used as no further sample processing was performed after labelling with reagents.

3.7 Serial dilutions.	State whether serial dilutions were performed on samples and note the dilution range and manner of testing. The fluorescence and/or scatter signal intensity would ideally be reported in standard units (see Section 4.3, 4.4) but arbitrary units can also be used. This data is best reported by plotting the recorded number events/concentration over a set period of time at different sample dilution. The median fluorescence intensity at each of the dilutions should also ideally be plotted on the same or a separate plot.	Serial dilution samples were measured at the same (initial) dilution and at the same concentration as matched stained controls and were recorded during the same experiment at the same imaging flow cytometer with acquisition settings similar to all other samples, including laser power and flow rate. Four times 4-fold dilution was performed by mixing 100 uL of (stained) sample with 300 uL of fPBS. Correlation analysis showed a linear correlation between the concentration of double-positive fluorescent EV (CFSE+Tetraspanin+) and dilution rate (R^2=0,93). Fluorescent intensities remained stable: ~113 ERF CFSE and ~32 ERF APC.
3.8. Detergent treated EV-samples	State whether samples were detergent treated to assess lability. If utilized, state what detergent was used, the end concentration of the detergent,	A 10% (v/v) Triton X-100 stock solution was made by dissolving 1 mL of TritonX-100 in 9 mL of fPBS. All samples (buffer alone, buffer plus reagents, unstained samples, single-stained samples, and double-stained samples) were treated with 20 µL of the Triton X-100 stock solution (final concentration: 0.5% (v/v) per test), followed by 30 minutes of incubation at room temperature in the dark prior to acquisition. Comparison of fluorescent concentrations in the PPP samples obtained before and after detergent lysis for CFSE+, Tetraspanin+, and

	and what the results were of the lysis.	CFSE+Tetraspanin+ regions showed ~31%, ~64% and ~94% reduction, respectively. For CD9+CD31+ EV, a ~93% reduction was observed after detergent lysis.
4.1 Trigger Channel(s) and Threshold(s).	The trigger channel(s) and threshold(s) used for event detection. Preferably, the fluorescence calibration (Section 4.3) and/or scatter calibration (Section 4.4) should be used in order to report the trigger channel(s) and threshold(s) in standardized units.	Based on unstained, single-stained and isotype control samples, detection for CFSE fluorescence was triggered with 488nm laser at full power (200 mW), detected in channel 2 (480-560 filter) at a threshold of 170 arbitrary units, equivalent to ~36 FITC ERF, deterimined using Spherotec Rainbow Calibration beads and the manufacturers calibration values. Similarly, APC fluorescence was triggered with 642 nm laser at full power (150 mW), detected in channel 5 (642-745 filter), at a threshold of 170 a.u., equivalent to ~6 ERF APC. BV421 fluorescence was triggered with 405 nm laser at full pwer (120 mW), detected in channel 1 (435-505 nm filter), at a threshold of 110 a.u., equivalent to ~678 C30 ERF.
4.2 Flow Rate / Volumetric quantification.	State if the flow rate was quantified/validated and if so, report the result and how they were obtained.	Flow speed was monitored during acquisition and acquisition was started when flow speed was between 43.5 - 43.7 mm/sec.<br>Typically, with the IFCM set at 'low speed, high sensitivity', ~0.8 uL of sample was measured in the time span of 180 seconds.

4.3 Fluorescence Calibration.	State whether fluorescence calibration was implemented, and if so, report the materials and methods used, catalogue numbers, lot numbers, and supplied reference units for the standards. Fluorescence parameters may be reported in standardized units of MESF, ERF, or ABC beads. The type of regression used, and the resulting scatter plot of arbitrary data vs standard data for the reference particles should be supplied.	Arbitrary BV421, CFSE and APC fluorescence intensities were converted to ERF units using 500 nm Rainbow Calibration Particles (RCP-05-5, lot AL01, Spherotech) with known Equivalent number of Reference Fluorophores (ERF) values for C30/FITC/APC. For each detection channel, the MFI of each fluorescent peak (blanc peaks were omitted from the regression analysis) from the four bead populations (1 blanc – 3 fluorescent) were measured, and a linear regression analysis was performed of the log(10) of these values against the log(10) of the known ERF values. The resulting equations were used to convert BV421/CFSE/APC fluorescent intensities into ERF units.
4.4 Light Scatter Calibration.	State whether and how light scatter calibration was implemented. Light scatter parameters may be reported in standardized units of nm2, along with information required to reproduce the model.	Light scattering signals were fitted with Mie theory using a previously described model. The BF detector was modelled as a forward scattered light detector collecting light using a lens with a numerical aperture (NA) of 0.9, which corresponds to the NA of the 60x objective. The center wavelength of brightfield detection was 618.5 nm. The SSC detector was modelled as a detector that is placed perpendicular to the propagation direction of the laser beam. The NA of the collection lens was 0.9 and the wavelength was 785.0 nm. PS beads were modelled as solid spheres with a refractive index (n) of 1.5885 for a wavelength of 618.5 nm (brightfield) and 1.5783 for a wavelength of 785.0 nm (SSC). EVs were modelled as core-shell particles with a core refractive index of 1.38, shell refractive index of 1.48 and a shell thickness of 6 nm for both wavelengths as the dispersion relation for the core and shell of EVs is unknown. Beads were

		measured in water, and EVs in PBS. Therefore, the refractive indices of PBS and water were assumed to be 1.3345 and 1.3325, respectively, at a wavelength of 618.5 nm (BF) and 1.3309 and 1.3289, respectively, at a wavelength of 785.0 nm (SSC).<br>Effective scattering cross sections of the calibration beads were calculated by integrating the amplitude scattering matrix elements over 576 collection angles. Data and theory were log10-transformed to scale the data onto the theory using a least-square-fit.
5.1 EV diameter/surface area/volume approximation.	State whether and how EV diameter, surface area, and/or volume has been calculated using FC measurements.	BF and SSC data of the PS beads were scaled onto Mie theory, resulting in a scaling factor (F) of 1.3518 and a coefficient of determination (R2) of 0.00 for the BF detector and a scaling factor of 8.405 and an R2 of 0.91 for the SSC detector. For the SSC detector, the theoretical model indicated a plateau between ~400 to ~800 nm, which translates into a low resolution when determining EV sizes based on SSC intensities within this region. The highest dynamic range was observed up to 400 nm - corresponding to a value of 900 a.u. SSC intensity.
5.2 EV refractive index approximation.	State whether the EV refractive index has been approximated and how this was done.	EV refractive index has not been approximated in this work - for Mie theory application, EVs were modelled as core-shell particles with a core refractive index of 1.38,<br>shell refractive index of 1.48 and a shell thickness of 6 nm for both wavelengths as the dispersion relation for the core and shell of EVs is unknown.
5.3 EV epitope number approximation.	State whether EV epitope number has been approximated, and if so, how it was approximated.	Other than conversion of fluorescent intensities into standardized units (ERF), no EV epitope numbers have been approximated in this work.

6.1 Completion of MIFlowCyt checklist.	Complete MIFlowCyt checklist criteria 1 to 4 using the MIFlowCyt guidelines. Template found at www.evflowcytometry.org.	The MIFlowCyt checklist v1.0.0 has been completed and attached in the Supplementary Information.
6.2 Calibrated channel detection range	If fluorescence or scatter calibration has been carried out, authors should state whether the upper and lower limits of a calibrated detection channel were calculated in standardized units. This can be done by converting the arbitrary unit scale to a calibrated scaled, as discussed in Section 4.3 and 4.4, and providing the highest unit on this scale and the lowest detectable unit above the unstained population. The lowest unit at which a population is deemed 'positive' can be determined a variety of ways, including reporting the 99th percentile measurement	The lower fluorescence threshold for Ch01 (BV421), Ch02 (CFSE), and Ch05 (APC) was set at 110, 170, and 170 a.u., respectively. These values were obtained by analysing blanc-fluorescent Rainbow Calibration Particles (RCP-05-5, lot AL01, Spherotech), unstained PPP samples, and isotype control PPP samples.<br>When scaled to ERF units, these values translated to 677.71 / 35.40 / 6.40 ERF, respectively.<br><br>Upper fluorescent limits (high-end gating cut-off) for Ch01 (BV421), Ch02 (CFSE), and Ch05 (APC) was set at 100.000, 50.553, and 10.302 a.u., respectively. These gating cut-offs were determined to encompass all obtained fluorescent events.<br>When scaled to ERF units, these values translated to 112,201 / 3776 / 123 ERF, respectively.

	unit of the unstained population for fluorescence. The chosen method for determining at what unit an event was deemed positive should be clearly outlined.	
6.3 EV number/concentration.	State whether EV number/concentration has been reported. If calculated, it is preferable to report EV number/concentration in a standardized manner, stating the number/concentration between a set detection range.	Detected concentrations of fluorescent EV are described in detail in the manuscript. All concentrations reported were obtained between the calibrated detection ranges for each channel, as described above.
6.4 EV brightness.	When applicable, state the method by which the brightness of EVs is reported in standardized units of scatter and/or fluorescence.	EV brightness was calculated for all fluorescent populations analyzed and described in the work; MFI values were converted into standardized-ERF values.<br>For CFSE+Tetraspanin+ EV measured in the PPP samples, we observed a mean EV brightness of 119.57 ERF (range 99.6-156) for CFSE and 65.33 ERF (range 61.3-69.8) for APC.<br>For CD9+CD31+ EV measured in the PPP samples, we observed a mean EV brightness of ~7,620 (range 3,640 – 9,240) and 20.4 (range 15 – 27.9) for BV421 and APC, respectively.

7.1. Sharing of data to a public repository.	Provide a link to the experimental data in a public data repository.	IFCM files can be obtained by contacting the corresponding author.

Supplementary Table 1 – Framework representing the Minimal Information about a Flow Cytometry (FC) experiment to allow standardized EV-FC-specific reporting (MIFlowCyt-EV template), as recommended by the Minimum Information for Studies of EVs (MISEV).

Requirement	Please Include Requested Information
1.1. Purpose	To develop a protocol for the direct measurement of Extracellular Vesicles (EV) in unprocessed (human) plasma samples.
1.2. Keywords	Unprocessed Human Plasma; Extracellular Vesicles; Imaging Flow Cytometry; Quantify; Phenotype; Diagnostic Platform
1.3. Experiment variables	Platelet-poor plasma (PPP) samples from 5 healthy individuals and/or six week old male C57BL/6J (JAX,GSP) mice (Jackson Labs, Bar Harbor, ME) were stained with CFDA-SE, anti-tetraspanin antibodies (CD9, CD63, CD81) and CD31, and measured with Imaging Flow Cytometry (IFCM).
1.4. Organization name and address	Erasmus Medical Center, University Medical Center Rotterdam, The Netherlands.<br>Wytemaweg 80, 3015 CN, Rotterdam
1.5. Primary contact name and email address	Wouter W. Woud, wouterwwoud@gmail.com
1.6. Date or time period of experiment	2020 - 2021
1.7. Conclusions	Imaging Flow Cytometry (IFCM) can be used to identify, quantify and phenotype fluorescently tagged EV ≤240 nm in **unprocessed** (human) plasma samples.
1.8. Quality control measures	The instrument calibration tool ASSIST® was used upon each startup to optimize performance and consistency between experiments. Additionally, commercially available mixtures of FITC-fluorescent polystyrene beads of known sizes (Megamix-Plus FSC – 900, 500, 300 and 100 nm, and Megamix-Plus SSC – 500, 240, 200, 160 nm), as well as Rainbow Calibration Particles (RCP-05-5, lot AL01, Spherotech), were used in calibrating and standardization of the IFCM platform.
2.1.1.1. (2.1.2.1., 2.1.3.1.) Sample description	Platelet-poor plasma (PPP) obtained from 5 healthy individuals was used in this study. From each of the 5 healthy individuals, 12 mL of blood was collected (one drawing) into two BD Vacutainer® K3-EDTA-coated collection tubes (BD Biosciences, San Jose, USA). Whole blood was centrifuged (Heraeus Multifuge 1S) at 1910 x g for 10 minutes at room temperature. The plasma layer was then collected - leaving ~1 mm of plasma above the buffy coat - and centrifuged (Heraeus Fresco) at 16,000 x g for 10 minutes at room temperature. The resulting PPP was divided into 700-µL aliquots in cryovials containing 28 µL of a 25x concentrated protease inhibitor cocktail solution (4% v/v) (cOmplete Protease inhibitor cocktail tablets, Roche, Mannheim, Germany) according to the manufacturers' instructions and stored at -80 °C. Additionally, PPP was generated from mice. All the procedures and animal housing conditions were carried out in strict accordance with current EU legislation on animal experimentation and were approved by the Institutional Committee for Animal Research (DEC protocol EMC No. AVD101002016635). Six weeks male C57BL/6J (JAX,GSP) mice (Jackson Labs, Bar Harbor, ME) were housed in Erasmus MC animal facility and housed in groups of 2-3/cage. They were maintained on a 12:12 h light-dark cycle and allowed ad libitum access to water and standard rodent food. The mice

	were anesthetized and blood (approximately 0.8 mL) was collected via the left ventricle using a 23-25 gauge needle. To ensure euthanasia of the animal post-procedure, mice were killed by cervical dislocation.
2.1.1.2. Biological sample source description	See above
2.1.1.3. Biological sample source organism description	Healthy human individuals – 2 male, 3 female, age range 31 – 56 (mean 43,4).<br>Mouse – see above.
2.1.2.2. Environmental sample location	NA
2.3. Sample treatment description	Bloods were drawn, processed and stored as described above. For staining, 30 uL of PPP was added to a pre-defined volume of fPBS (dependant on the volume of mAb staining - total volume after mAb addition was set at 130 µL): 12.5 uL of the stock solutions containing mAbs labelled with –APC and 5 µL of the stock solutions containing mAbs labeled with –BV421 were added, resulting in the following concentrations used per test: anti-CD9 – 2.5 ng, anti-CD63 – 83 ng, anti-CD81 – 83 ng, anti-CD31 (anti-human) – 1 ng, anti-CD31 (anti-mouse) – 40 ng per test. Equivalent amounts of isotype control was used for each antibody.<br>Samples were then incubated overnight at 4 °C to ensure optimal saturation of the available EV epitopes; this incubation time was determined by adding the anti-tetraspanin antibody mix to fPBS and PPP samples and performing acquisition at set intervals (1/3/6 hours and O/N).<br>CFDA-SE labeling was performed on the day of data acquisition by adding 100 µL of the 50 µM CFDA-SE stock solution to the samples, followed by 30 minutes of incubation at room temperature in the dark. Control samples not stained with CFDA-SE were incubated with 100 µL fPBS instead. All samples were brought to a total volume of 380 µL using fPBS before IFCM measurements.
2.4. Fluorescence reagent(s) description	The monoclonal antibodies (mAbs) used to stain human PPP were anti-CD9–APC, clone HI9a (6 µg/mL, BioLegend, San Diego, USA); anti-CD63–APC, clone H5C6 (200 µg/mL, BioLegend); and anti-CD81–APC, clone 5A6 (200 µg/mL, BioLegend. Human and mouse PPP were both stained with anti-human CD31–BV421, clone WM-59 (50 µg/mL, BioLegend) and anti-mouse CD31-APC, clone 390 (200 µg/mL, BioLegend). Isotype controls used were IgG1,k-BV421, clone MOPC-21 (100 µg/mL, BioLegend); IgG1,k-APC, clone MOPC-21 (200 µg/mL, BioLegend); and IgG2a,k-APC, clone RTK2758 (200 µg/mL, BioLegend).
3.1. Instrument manufacturer	LUMINEX
3.2. Instrument model	ImageStream^X MkII
3.3. Instrument configuration and settings	The ISx was equipped with three objectives (20x/40x/60x) and 1 CCD camera. All data were acquired using the 60x objective (numerical aperture of 0.9 – pixel area of 0.1 µm^2) with fluidics settings set to "low speed/high sensitivity".<br>We adjusted the default core size of 7 µm to 6 µm using the "Defaults Override" option within INSPIRE software (version 200.1.681.0), as recommended by

<table>
<tr><td></td><td>the manufacturer. Data were acquired over three minutes for standardization among samples with the autofocus setting activated and the “Remove Speedbead” option unchecked. BV421 fluorescence signals were collected in channel 1 (435–505-nm filter), CFSE signals in channel 2 (480–560-nm filter) and APC signals in channel 5 (642–745-nm filter). Channel 4 was used as the brightfield channel, and channel 6 (745–780-nm filter) was used for SSC detection. Excitation lasers were set as follows: 405 nm: 120 mW, 488 nm: 200 mW, 642 nm: 150 mW, and 775 nm (SSC): 1.25 mW. Particle enumeration was achieved through the advanced fluidic control of the ISx coupled with continuously running SBs and application of the “objects/mL” feature within the ISx Data Exploration and Analysis Software (IDEAS®).</td></tr>
<tr><td>4.1. List-mode data files</td><td>IFCM files can be obtained by contacting the corresponding author.</td></tr>
<tr><td>4.2. Compensation description</td><td>Fluorescent events from singly stained PPP samples were used in the setting of compensation matrices (to compensate for spectral overlap between fluorochromes) such that straight fluorescent populations were obtained when depicted in scatterplots. The following compensation matrix was established for all fluorophores used in this manuscript:
<table>
<tr><th></th><th>Ch01</th><th>Ch02</th><th>Ch03</th><th>Ch04</th><th>Ch05</th><th>Ch06</th></tr>
<tr><td>Ch01</td><td>1</td><td>0,07</td><td>0</td><td>0,022</td><td>0,025</td><td>0</td></tr>
<tr><td>Ch02</td><td>0,111</td><td>1</td><td>0</td><td>0,022</td><td>0,02</td><td>0</td></tr>
<tr><td>Ch03</td><td>0</td><td>0</td><td>1</td><td>0,026</td><td>0</td><td>0</td></tr>
<tr><td>Ch04</td><td>0</td><td>0</td><td>0</td><td>1</td><td>0</td><td>0</td></tr>
<tr><td>Ch05</td><td>0,002</td><td>0,02</td><td>0</td><td>0,028</td><td>1</td><td>0</td></tr>
<tr><td>Ch06</td><td>0,013</td><td>0,035</td><td>0</td><td>0,034</td><td>0</td><td>1</td></tr>
</table></td></tr>
<tr><td>4.3. Data transformation details</td><td>Arbitrary BV421, CFSE and APC fluorescence intensities were converted to ERF units using 500 nm Rainbow Calibration Particles (RCP-05-5, lot AL01, Spherotech)
with known Equivalent number of Reference Fluorophores (ERF) values for C30/FITC/APC. For each detection channel, the MFI of each peak from the four bead
populations (1 blanc – 3 fluorescent) were measured, and a linear regression analysis was performed of the log(10) of these values against the log(10) of the known
ERF values. The resulting equations were used to convert BV421/CFSE/APC fluorescent intensities into ERF units.</td></tr>
<tr><td>4.4.1. Gate description</td><td>The lower fluorescence threshold for Ch01 (BV421), Ch02 (CFSE), and Ch05 (APC) was set at 110, 170, and 170 a.u., respectively. These values were obtained by analyzing blanc-fluorescent Rainbow Calibration Particles (RCP-05-5, lot AL01, Spherotech), unstained PPP samples, and isotype control PPP samples.
When scaled to ERF units, these values translated to 1397.171 / 38.40 / 28.03 ERF, respectively.

Upper fluorescent limits (high-end gating cut-off) for Ch01 (BV421), Ch02 (CFSE), and Ch05 (APC) was set at 100.000, 50.553, and 10.302 a.u., respectively.</td></tr>
</table>

	These gating cut-offs were determined to encompass all obtained fluorescent events.<br>When scaled to ERF units, these values translated to 89125 / 3656 / 133 ERF, respectively.
4.4.2. Gate statistics	Median Fluorescent Intensity (MFI) – Count – Objects/mL
4.4.3. Gate boundaries	See above

Supplemental Table 2 – Checklist representing the Minimal Information about a Flow Cytometry (FC) experiment to allow standardized EV-FC-specific reporting (MIFlowCyt-EV checklist), as recommended by the Minimum Information for Studies of EVs (MISEV).

**Control type**	**Rationale**
PBS	Blanc - Background control
PBS + mAbs	mAb mediated background control
PBS + Isotypes	Isotype mediated background control
Unstained sample	Autofluorescence of unstained sample
Sample + Single stain	Fluorescence compensation purpose
Sample + Isotype	Unspecific binding of antibodies used
Sample + Double stain	***Multiparameteric detection of sample of interest***
Sample + Double stain + Detergent Treatment	Confirmation that detected events are of biological nature

Supplementary Table 3 - Control types and the rationale for their use. Each control listed above is essential for the multiparametric detection of human plasma-derived single EV.

Parameter	Settings
Magnification:	60x
Lasers:	405nm – 488nm – 642nm - SSC (785 nm)
Voltage:	120mW – 200mW – 150mW – 1.25mW
Fluidics:	Low Speed & High Sensitivity
Autofocus:	ON
Remove Speedbead:	Unchecked
Core Widt:	6 um (Override)
Acquisition time:	3 minutes

Supplementary Table 4 - Acquisition parameter settings for the multiparametric detection of single EV in human plasma samples using the ISx MKII imaging flow cytometer. Lasers were turned on as applicable for each experiment. SSC: Side Scatter.
