# Supplementary material for: An imaging flow cytometry-based methodology for the analysis of single extracellular vesicles in unprocessed human plasma
Source: Commun Biol. 2022 Jun 29;5:633. doi: 10.1038/s42003-022-03569-5 (PMC9243126; doi:10.1038/s42003-022-03569-5)
Supplement: Supplementary file 3 — Description of Additional Supplementary Files [file 42003_2022_3569_MOESM3_ESM.pdf]

Description of Additional Supplementary Files

File Name: Supplementary Data 1-8

Description: Source data underlying the figures as presented in the manuscript. Each tab contains the source data for their corresponding figure. Each column with a name corresponding to the fluorophores as described in the manuscript (e.g., CFSE, CD9, CD63, CD81, Tetraspanin (= anti-tetraspanin antibody mixture – comprised of anti-CD9, anti-CD63, and anti-CD81), and CD31) represents ‘objects/mL’ data as calculated by the IDEAS software.
