# Supplementary material for: An imaging flow cytometry-based methodology for the analysis of single extracellular vesicles in unprocessed human plasma
Source: Commun Biol. 2022 Jun 29;5:633. doi: 10.1038/s42003-022-03569-5 (PMC9243126; doi:10.1038/s42003-022-03569-5)
Supplement: Supplementary file 5 — Life Science Reporting Summary [file 42003_2022_3569_MOESM5_ESM.pdf]

nature portfolio

Corresponding author(s): Wouter W. Woud

Last updated by author(s): 18/02/22

Reporting Summary

Nature Portfolio wishes to improve the reproducibility of the work that we publish. This form provides structure for consistency and transparency in reporting. For further information on Nature Portfolio policies, see our Editorial Policies and the Editorial Policy Checklist.

Statistics

For all statistical analyses, confirm that the following items are present in the figure legend, table legend, main text, or Methods section.

n/a	Confirmed	
☐	☒	The exact sample size (*n*) for each experimental group/condition, given as a discrete number and unit of measurement
☐	☒	A statement on whether measurements were taken from distinct samples or whether the same sample was measured repeatedly
☐	☒	The statistical test(s) used AND whether they are one- or two-sided<br>*Only common tests should be described solely by name; describe more complex techniques in the Methods section.*
☐	☒	A description of all covariates tested
☐	☒	A description of any assumptions or corrections, such as tests of normality and adjustment for multiple comparisons
☐	☒	A full description of the statistical parameters including central tendency (e.g. means) or other basic estimates (e.g. regression coefficient) AND variation (e.g. standard deviation) or associated estimates of uncertainty (e.g. confidence intervals)
☒	☐	For null hypothesis testing, the test statistic (e.g. *F*, *t*, *r*) with confidence intervals, effect sizes, degrees of freedom and *P* value noted<br>*Give P values as exact values whenever suitable.*
☒	☐	For Bayesian analysis, information on the choice of priors and Markov chain Monte Carlo settings
☒	☐	For hierarchical and complex designs, identification of the appropriate level for tests and full reporting of outcomes
☒	☐	Estimates of effect sizes (e.g. Cohen's *d*, Pearson's *r*), indicating how they were calculated

Our web collection on statistics for biologists contains articles on many of the points above.

Software and code

Policy information about availability of computer code

Data collection	Imaging Flow Cytometry acquisition software: ISX Version 201.1.0.693
Data analysis	Imaging Flow Cytometry data analysis software: IDEAS Application Version 6.2.187.0<br>Data Analysis and Graph generation and statistical testing: R-Studio Version 4.0.2 (Open Source)

For manuscripts utilizing custom algorithms or software that are central to the research but not yet described in published literature, software must be made available to editors and reviewers. We strongly encourage code deposition in a community repository (e.g. GitHub). See the Nature Portfolio guidelines for submitting code & software for further information.

Data

Policy information about availability of data

All manuscripts must include a data availability statement. This statement should provide the following information, where applicable:

- Accession codes, unique identifiers, or web links for publicly available datasets
- A description of any restrictions on data availability
- For clinical datasets or third party data, please ensure that the statement adheres to our policy

The datasets generated and analyzed during the current study are available from the corresponding author on reasonable request.

Field-specific reporting

Please select the one below that is the best fit for your research. If you are not sure, read the appropriate sections before making your selection.

☒ Life sciences ☐ Behavioural & social sciences ☐ Ecological, evolutionary & environmental sciences

For a reference copy of the document with all sections, see nature.com/documents/nr-reporting-summary-flat.pdf

Life sciences study design

All studies must disclose on these points even when the disclosure is negative.

Sample size	The sample size of 5 healthy individuals was deemed sufficient for this 'proof of principle' study, in which we present an Imaging Flow Cytometry (IFCM)-based methodology to analyze single Extracellular Vesicles in unprocessed human PPP samples.
Data exclusions	No data was excluded throughout this study.
Replication	Reproducibility was verified by measuring two samples, each in the following manner: 1tube - 5 measurements (variability ~5%), and 5 tubes - 1 measurement each (variability ~15%)
Randomization	All human and mouse samples measured/used in this study were obtained from healthy individuals - therefore no randomization of samples was performed.
Blinding	Blinding was not relevant; all samples were obtained from healthy individual and no groups were formed.

Reporting for specific materials, systems and methods

We require information from authors about some types of materials, experimental systems and methods used in many studies. Here, indicate whether each material, system or method listed is relevant to your study. If you are not sure if a list item applies to your research, read the appropriate section before selecting a response.

Materials & experimental systems

n/a	Involved in the study	
☐	☒	Antibodies
☒	☐	Eukaryotic cell lines
☒	☐	Palaeontology and archaeology
☐	☒	Animals and other organisms
☐	☒	Human research participants
☒	☐	Clinical data
☒	☐	Dual use research of concern

Methods

n/a	Involved in the study	
☒	☐	ChIP-seq
☐	☒	Flow cytometry
☒	☐	MRI-based neuroimaging

Antibodies

Antibodies used

Anti-Human:
APC anti-CD9, Supplier: Biolegend, Catalogue Number: 312108, Clone name: HI9a, Lot Number: B293102
APC anti-CD63, Supplier: Biolegend, Catalogue Number: 353008 Clone name: H5C6, Lot Number: B276014
APC anti-CD81, Supplier: Biolegend, Catalogue Number: 349510, Clone name: 5A6, Lot Number: B296062
IgG1,k Isotype Control - APC, Supplier: Biolegend, Catalogue Number: 400119, Clone name: MOPC-21, Lot Number: B201922

BV421 anti-CD31, Supplier: Biolegend, Catalogue Number: 303124, Clone name: WM-59, Lot Number: B298418
IgG1,k Isotype Control - BV421 Supplier: Biolegend, Catalogue Number: 400158, Clone name: MOPC-21, Lot Number: B285247

Anti-Mouse:
APC anti-CD31, Supplier: Biolegend, Catalogue Number: 102409, Clone name: 390, Lot Number: B307911
IgG2a,k Isotype Control - APC Supplier: Biolegend, Catalogue Number: 400511, Clone name: RTK2758, Lot Number: B316055

Validation

Anti-Human:
CD9 anti-human antibody: https://www.biolegend.com/nl-nl/products/apc-anti-human-cd9-antibody-15072
CD63 anti-human antibody: https://www.biolegend.com/nl-nl/products/apc-anti-human-cd63-antibody-7486
CD81 anti-human antibody: https://www.biolegend.com/nl-nl/products/apc-anti-human-cd81-tapa-1-antibody-10228
APC Mouse IgG1,k Isotype Ctrl Antibody: https://www.biolegend.com/nl-nl/products/apc-mouse-igg1-kappa-isotype-ctrl-1404

CD31 anti-human antibody: https://www.biolegend.com/nl-nl/products/brilliant-violet-421-anti-human-cd31-antibody-8588
BV421 Mouse IgG1,k Isotype Ctrl Antibody: https://www.biolegend.com/nl-nl/products/brilliant-violet-421-mouse-igg1-kappa-isotype-ctrl-7194

CD31 anti-mouse antibody: https://www.biolegend.com/nl-nl/products/apc-anti-mouse-cd31-antibody-118
APC Mouse IgG2a,k Isotype Ctrl Antibody: https://www.biolegend.com/nl-nl/products/apc-rat-igg2a-kappa-isotype-ctrl-1838

Animals and other organisms

Policy information about studies involving animals; ARRIVE guidelines recommended for reporting animal research

Laboratory animals	Six weeks old male C57BL/6J (JAX, GSP) mice (Jackson Labs, Bar Harbor, ME)
Wild animals	No wild animals were used in this study
Field-collected samples	This study did not involve samples collected from the field
Ethics oversight	All the procedures and animal housing conditions were carried out in strict accordance with current EU legislation on animal experimentation and were approved by the Institutional Committee for Animal Research (DEC protocol EMC No. AVD101002016635)

Note that full information on the approval of the study protocol must also be provided in the manuscript.

Human research participants

Policy information about studies involving human research participants

Population characteristics	Platelet-poor plasma was generated from 5 healthy human individuals: 2 males, 3 females, average age: 43.3 years, age range: 31-56 years.
Recruitment	All individuals provided written informed consent.
Ethics oversight	Sample collection and processing was approved by the Medical Ethical Review Board (MERB number MEC-2018-1623) and conducted in accordance with the Declaration of Helsinki.

Note that full information on the approval of the study protocol must also be provided in the manuscript.

Flow Cytometry

Plots

Confirm that:

☒ The axis labels state the marker and fluorochrome used (e.g. CD4-FITC).

☒ The axis scales are clearly visible. Include numbers along axes only for bottom left plot of group (a 'group' is an analysis of identical markers).

☒ All plots are contour plots with outliers or pseudocolor plots.

☒ A numerical value for number of cells or percentage (with statistics) is provided.

Methodology

Sample preparation	12 mL of blood was collected (one drawing) from each individual into two BD Vacutainer® K3-EDTA-coated collection tubes (BD Biosciences, San Jose, USA). Whole blood was centrifuged (Heraeus Multifuge 1S) at 1910 x g for 10 minutes at room temperature. The plasma layer was then collected - leaving ~1 mm of plasma above the buffy coat - and centrifuged (Heraeus Fresco) at 16,000 x g for 10 minutes at room temperature in 1mL aliquots using Safe-Lock Eppendorf tubes (Eppendorf AG, Hamburg, Germany). The resulting platelet-poor plasma (PPP) was first pooled before being divided into 700-µL aliquots in cryovials containing 28 µL of a 25x concentrated protease inhibitor cocktail solution (4% v/v) (cOmplete Protease inhibitor cocktail tablets, Roche, Mannheim, Germany) according to the manufacturers' instructions and stored at -80 °C
Instrument	ImageStream MKII instrument (ISx; Luminex, Texas, USA)
Software	Imaging Flow Cytometry acquisition software: ISX version 201.1.0.693<br>Imaging Flow Cytometry data analysis software: IDEAS version 6.2.187.0<br>Data analysis, graph generation and statistical testing: R-Studio version 4.0.2 (Open Source)
Cell population abundance	No cell sorting (or measurement) was performed in this work. In order to detect sub-micron sized EVs, we performed both size and fluorescence calibrations for the IFCM - both described in detail in the manuscript. Additionally, to verify EV measurements we applied the following controls: Buffer only / Buffer + reagents / Unstained PPP / single-stained PPP/ isotype controls. Detergent treatment was used to disrupt the lipid bilayer of EVs and thereby remove EV signals from measurements. Serial dilution of double-stained samples proved the detection and analysis of single EVs (as opposed to multiplet detection).
Gating strategy	The gating strategy is extensively described in the manuscript. in brief, fluorescent events =< 400 nm were identified after calibration of SSC intensities (following Mie theory and calculations). These events were checked for coincidence detection, and events representing multiple sub-micron sized particles (2 or more spots) or fluorescent events that did not occupy the same location on the pixel grid were excluded from analysis. The remaining events were analyzed based on their fluorescent

signals for the mAbs used.

Depending on the fluorescent probe used, the following lower-and upper gating areas were used:
Ch01 (BV421 signals) 110 a.u. (lower limit) and 100,000 a.u. (upper limit)
Ch02 (CFSE signals) 170 a.u. (lower limit) and 50.553 a.u. (upper limit)
Ch05 APC signals) 170 a.u. (lower limit) and 10.302 a.u. (upper limit)

After calibration of fluorescent intensities from arbitratry units into Equivalent numbers of Reference Fluorophores (ERF), these value were scaled to:
Ch01 (BV421 signals) 677.71 ERF (lower limit) and 112,201 ERF (upper limit)
Ch02 (CFSE signals) 35.40 ERF (lower limit) and 3776 ERF (upper limit)
Ch01 APC signals) 6.40 ERF (lower limit) and 123 ERF (upper limit)

☒ Tick this box to confirm that a figure exemplifying the gating strategy is provided in the Supplementary Information.
